# Current Dental Literature

**Published:** 1890-10

**Authors:** Geo. W. Williams

**Affiliations:** Richmond, Ind.


ARTICLE VI.

### CURRENT DENTAL LITERATURE.

BY GEO. W. WILLIAMS, RICHMOND, IND.

Current dental literature, as an educating power, is no mean factor, especially to those in the profession. It is a question whether it is not the best means in use. Books are more elaborate, colleges more exacting, but for variety, range and freshness—in its influence to produce a gradual awakening through its continual presence—current dental literature exerts the greatest force. Books and colleges give more elaborate theories, invite us to more sumptuous feasts and impress us with greater dignity: they make us more professional, round us out with greater completeness, and give greater definiteness to our knowledge. Current dental literature, by its "line upon line, and precept upon precept, here a little and there a little," by the varied stand-points to which it attracts us, and by its practical appilcation to the minutiæ of every day life, imperceptibly moulds us. It prepares us for our books and colleges, and follows us closely afterwards, ever doing us good.

There are now in existence in the literary field of medicine, dentistry and allied sciences something over 120,000 bound volumes, and about 240,000 pamphlets, making a total of more than one-third of a million of distinct and separate publications. When we go to count books by the million, it is very much the same as money by the million —not one in a thousand of us has any adequate conception of what the language means. To make a simple calculation, I will assume that we enter our professional career at the age of twenty-five. By a very liberal allowance, I will give you forty years, of 300 working days each, in which to become learned, famous and wealthy. In order that you might read the entire mass of existing professional literature, it would be necessary for you to read ten bound volumes each day and twenty pamphlets; one volume and two pamphlets each hour, providing you worked upon the "ten hour system." But this is only the beginning of sorrows, for the clatter and bang of the professional press is never silent for a moment, and it is pouring its stream through the flood-gates of the market at the rate of 1,500 volumes and 2,500 pamphlets a year. This annual product is the result of the labors of 11,600 writers; and here as usual, the American profession comes to the front with 2,800 writers, exceeding by 200 the most prolific nation of Europe—which is France with all her colonies. So you would need to add five volumes and eight pamphlets each day, and even more than this, as the current volume swells. Let us pause before this prodigious mountain, composed of some of the rarest gems, much lumber and more offal. We have caught this extensive literature, or it has caught us. I scarcely know which. Vast as it is, however, all that is really valuable has been gleaned, and sifted, and condensed until we have it within a moderately small compass. So that, with reasonable industry, moderate means, and, what is of no less importance, good judgment in making selections, nothing that is intrinsically valuable,either as simply broadening the field of human knowledge, or as conductive to the highest interests of humanity need escape our attention. All progress is recorded through this medium, and the newest and freshest thought is always presented to the reading world—not only of our own profession, but with rare exceptions in everything. The vast swelling tide of thought and progress, of investigation and discovery, which is sweeping over the world in every department of science and art,is nowhere greater than in the dental profession; and it is our duty to keep abreast of this tide, that we may give our patients the benefit of the most recent and yet fully tried discoveries. In order to accomplish this, we must acquaint ourselves with the current literature of our profession, which will bring before us the thinkings, doings and revelations of the professional world. —*Dental Mirror*.

